# Sarcopenia, Muscle Mass and Protein Intake in Adults Older Than 65 Years After Earlier Bariatric Surgery

**DOI:** 10.1002/jcsm.13839

**Published:** 2025-06-05

**Authors:** Gabriël Eksteen, Tim Vanuytsel, Roman Vangoitsenhoven, Ann Mertens, Matthias Lannoo, Ellen De Leus, Bart Van der Schueren, Christophe Matthys

**Affiliations:** ^1^ Clinical and Experimental Endocrinology KU Leuven Leuven Belgium; ^2^ Translational Research in Gastrointestinal Disorders KU Leuven University Hospitals Leuven Leuven Belgium; ^3^ Department of Gastroenterology and Hepatology University Hospitals Leuven Leuven Belgium; ^4^ Department of Endocrinology University Hospitals Leuven Leuven Belgium; ^5^ Department of Abdominal Surgery University Hospitals Leuven Leuven Belgium

**Keywords:** bariatric surgery, body composition, dietary proteins, obesity, sarcopenia, skeletal muscle

## Abstract

**Background:**

Metabolic and bariatric surgery (MBS) is a proven treatment for obesity. Yet weight loss is accompanied by loss of muscle which may predispose to sarcopenia. The prevalence of low muscle mass in older adults after MBS remains unexplored, even though this group is more vulnerable to sarcopenia.

**Methods:**

This cross‐sectional study investigated sarcopenia and low muscle mass by comparing adults older than 65 years with previous MBS (BAR) to patients following nonsurgical obesity management (CON). A sample size of 100 was estimated from appendicular lean mass (ALM) in a similar study in younger adults. Patients were recruited from the University Hospitals Leuven Obesity Clinic, Belgium. Study assessments included dual‐energy X‐ray absorptiometry, handgrip, short battery of physical performance, blood sampling and self‐reported dietary intake. Sarcopenia was defined according to the European Working Group on Sarcopenia in Older People (EWGSOP1) criteria using obesity‐specific cut‐off points and sarcopenic obesity by the European Society for Enteral and Parenteral Nutrition (ESPEN) and the European Association of the Study of Obesity (EASO) consensus definition. Main endpoints were sarcopenia and ALM normalized to body mass index (%ALM/BMI). A multiple linear regression model was fitted to predict ALM.

**Results:**

We included 50 participants per group (male, BAR 40%, CON 35%). BAR participants were older (68.3 ± 3.2 years vs. 70.7 ± 3.9, *p* < 0.01), and more had diabetes (52% vs. 28%). BAR lost more bodyweight after MBS than CON following nonsurgical treatment (BAR 31.6 ± 9.5% vs. CON 12.1 ± 8.42%, *p* < 0.001). Fat free mass (FFM) was lower for BAR than for CON, but %ALM/BMI was not different (64.7 ± 18.1% vs. 62.6 ± 15.8, *p* = 0.53). Twenty percent to 56% of participants had low muscle mass, depending on sex and criterium, but only 3% met the criteria for sarcopenia and 9% for sarcopenic obesity. Protein intake tended to be higher in BAR than in CON (1.36 ± 0.36 g/kg FFM/day vs. 1.25 ± 0.27, *p* = 0.09). Most participants did not meet optimal protein intake recommendations after BMS nor for older adults in general. In the linear regression model, muscle mass increased with male sex, BMI, adiposity and protein intake and decreased with age, (adjusted *R* ^2^ 0.80). Neither BAR compared to CON nor surgery type or other clinical parameters influenced muscle mass.

**Conclusion:**

Older adults with previous MBS were not more likely to develop sarcopenia than older adults following nonsurgical treatment. Rather, age, adiposity and low protein intake lower muscle mass, predisposing to sarcopenia.

**Trial Registration:** clinicaltrials.gov identifier: NCT05582668.

## 1 Introduction

The world's population is ageing and gaining weight, creating a profound public health challenge. Obesity prevalence statistics are based on body mass index (BMI) and should be cautiously interpreted in older adults where muscle mass declines and fat mass increases. Notwithstanding, overweight prevalence in Europe is the highest in adults aged 65 to 74 years [1]. Simultaneously, sarcopenia is becoming a greater concern as the population ages. Sarcopenia and obesity can co‐occur, termed sarcopenic obesity [2]. This can be an insidious condition as sarcopenia is masked by obesity, yet there is significant crosstalk through shared risks and underlying mechanisms. The pathogeneses both of obesity and sarcopenia are characterized by low‐grade inflammation, insulin resistance and myosteatosis, leading to poorer morbidity and increased frailty [3].

Metabolic and bariatric surgery (MBS) is a proven treatment for obesity and typically results in 30% weight loss within the first year [4]. This rapid weight loss leads to an average fat free mass (FFM) loss of 7 kg [5]. Several studies have investigated the increased risk of sarcopenia after MBS but rarely include older adults, even though they are more vulnerable to the effects of lean tissue loss [6, 7, 8, 9]. While most MBS is performed in younger adults, the anatomical reconfiguration of the gastrointestinal tract could have repercussions into later life. Inadequacies of nutrient intake and absorption could accumulate to make older adults with previous MBS more prone to sarcopenia than their peers. In addition, protein intake is often low after MBS, due to early satiety, taste changes, and food intolerances [10]. Inadequate protein intake can exacerbate the loss of lean mass after MBS, although reports are inconsistent [10].

The prevalence of low muscle mass and sarcopenia in older adults after MBS remains unexplored. Sarcopenia may be more prevalent compared to older adults with obesity who were treated nonsurgically. Alternatively, greater weight loss may enable more physical activity compared to patients who remain obese, improving physical performance and muscle mass. Additionally, weight loss after MBS results in improved glycaemic status and inflammation, which in turn may reduce the risk of sarcopenia [11]. The aim of this study was to investigate sarcopenia and low muscle mass in adults older than 65 years who previously underwent MBS, compared to patients following nonsurgical obesity treatment.

## 2 Methods

### 2.1 Study Design and Setting

This was a cross‐sectional study comparing patients older than 65 years with previous MBS (BAR group) to a group of patients older than 65 years who received nonsurgical obesity management (CON group). The primary endpoint was the prevalence of sarcopenia, and the secondary endpoint was appendicular lean mass (ALM), a measure of skeletal muscle mass (SMM). Exposure to MBS was identified retrospectively, and the outcomes were assessed cross‐sectionally. This single‐centre study was performed at the University Hospitals Leuven Obesity Clinic in Leuven, Belgium. The sample size was based on a study which investigated sarcopenia in 60 women after MBS in comparison to a matched nonsurgical group where ALM index (ALM/height^2^) was 6.88 ± 1.0 kg/m^2^ and 7.58 ± 1.1, respectively [6]. A priori sample size calculation in G*Power 3.9.1.7 with a one‐tailed alpha of 0.05, power of 0.95, allocation of 1:1 and effect size of 0.67 resulted in a sample size of 50 participants per group.

Participants were recruited from an ongoing clinical patient registry. Patients were prescreened and contacted by telephone to participate in the study. Recruitment continued through convenience sampling until the sample size was reached, commencing in December 2022 and concluding in July 2024. The study was registered with clinicaltrials.gov (NCT05582668). Reporting is aligned to the Strengthening the Reporting of Observational Studies in Epidemiology (STROBE) guidelines [12].

### 2.2 Participants

We included patients from 1 year after MBS but did not limit the maximum time lapsed, to include the time since surgery as an effect modifier. We included adults older than 65 years who consented to participate in the study. For BAR, patients with previous MBS were included, except for gastric banding. For CON, we included patients assessed in the obesity clinic, without indication of MBS or patients chose not to undergo surgery. Exclusion criteria included diseases known to cause disease‐associated sarcopenia such as advanced organ failure, recent or current cancer, debilitating neuromuscular conditions, inability to mobilize without a wheelchair and weight above 130 kg, which precludes dual‐energy X‐ray absorptiometry (DXA) assessment.

### 2.3 Clinical and Anthropometric Measurements

All study assessments occurred on 1 day after an overnight fast. Weight and height were measured with calibrated equipment and medical history was self‐reported. Type and date of MBS and weight history were retrieved form electronic medical records. Baseline weight was weighted before MBS or weighted on the first obesity clinic assessment for CON. For the secondary surgery, the weight before the last surgery (mostly RYGB) was considered as baseline. Weight loss was calculated as percentage total weight loss (TWL) and as percentage excess weight loss (EWL), where excess weight corresponds to additional weight above an ideal body weight (IBW) at a BMI of 25 kg/m^2^. Both TWL and EWL were calculated from baseline to treatment nadir.

### 2.4 Body Composition Measurements

Body composition was measured by DXA using a Horizon scanner (Hologic, USA) and in some instances a Lunar Prodigy scanner (GE Healthcare, USA). Lunar values were adjusted for systematic differences between the equipment [13]. SMM was estimated based on a recently developed model, including older adults and obesity [14]. To express muscularity relative to adiposity, we used ALM divided by BMI, an indicator developed for the Foundation for the National Institutes of Health Sarcopenia Project [15]. We expressed this as a percentage (%ALM/BMI) for ease of use. Obesity was defined by fat percentage above 43% for females and 31% for males, based on age ranges recommended by the European Society for Clinical Nutrition and Metabolism/European Association for the Study of Obesity (ESPEN/EASO) sarcopenic obesity consensus statement [2]. We estimated the resting energy expenditure (REE) using the Mifflin–St Jeor Equation, which shows less bias than other equations after MBS [16].

### 2.5 Blood Biochemistry

Blood samples were collected using BD Vacutainer® tubes (Becton, Dickinson and Company, Franklin Lakes, USA). Whole blood was analysed for haemoglobin, platelet count and HbA1C. Blood serum was analysed for creatinine, albumin, aspartate transaminase (AST), alanine transaminase (ALT), cystatin C, blood glucose, insulin, C‐reactive protein (CRP), 25‐hydroxyvitamin D and zinc. Plasma was analysed for blood glucose. Blood analysis was conducted by the UZ Leuven clinical laboratory using standardized protocols. For reporting CRP as a measure of chronic low‐grade inflammation, values above 10 mg/L were excluded as probable acute inflammation. HOMA‐IR (Homeostatic Model Assessment for Insulin Resistance) was calculated except for participants who were nonfasting or prescribed with exogenous insulin. To predict the presence of metabolic dysfunction associated liver disease (MAFLD), we used the *NAFLD‐Fibrosis score* and adjusted for severe obesity as previously done [17]. Scores below −1.1455 are indicative of no fibrosis, while scores greater than 0.676 predict fibrosis. We calculated the *sarcopenic index* from cystatin C and creatinine, as a validated biomarker of sarcopenia [18].

### 2.6 Physical Activity, Gastrointestinal Symptoms and Quality of Life

Physical activity was self‐reported using the International Physical Activity Questionnaire, short form (IPAQ‐SF) [19]. Gastrointestinal symptoms were measured using the gastrointestinal symptom rating scale (GSRS), where higher scores indicate more severe symptoms [20]. Health‐related quality of life (HRQoL) was assessed using the EQ‐5D‐5L, and index scores were calculated using a Belgian value set [21].

### 2.7 Physical Performance, Muscle Strength and Sarcopenia Assessments

Physical performance was measured by the short physical performance battery (SPPB), and gait speed was reported separately as an additional measure of physical performance [22]. Skeletal muscle strength was measured by handgrip strength using a Jamar dynamometer (Performance Health, Warrenville, USA), using the Southampton standardized protocol [23]. Handgrip strength was interpreted according to the European Working Group on Sarcopenia in Older People consensus guidelines (EWGSOP1) which provides reference ranges per BMI category [24]. Muscle strength was also assessed by sit‐to‐stand test, a part of the SPPB. Sarcopenia was defined by the EWGSOP1 criteria which provide obesity‐specific cut‐off points [24]. Sarcopenic obesity was assessed according to the ESPEN/EASO criteria [2].

### 2.8 Dietary Intake Assessment

Dietary intake was measured using two nonconsecutive 24‐h dietary recalls, one during the study visit and one unannounced by telephone, following the automated multiple‐pass method [25]. Dietary recalls were completed by the first author, a dietitian or two researchers under his direct supervision. Portion sizes were estimated using sketches of portions sizes, household measures or branded items. Records were analysed using the Belgian food composition database (version 2020, Nubel, Brussels, Belgium), and missing values were extracted from the Dutch Food composition database (NEVO). The total daily energy intake (kcal) and macronutrient intake were calculated as the average of 2 days. To assess adequacy of protein intake for older adults, we used cut‐off points of 1.0 g/kg/day actual bodyweight and 1.6 g/kg/day FFM [26, 27]. Protein intake after MBS was compared to recommendations from clinical guidelines of at least 60 g/day and up to 1.5 g/kg IBW [28].

### 2.9 Statistical Analysis

For comparisons between BAR and CON, an independent sample student *t*‐test was used for continuous data and chi‐square for categorical variables. When comparing subsets, normality was evaluated by the Shapiro–Wilk test and by visually inspecting the data distribution. Values are reported at means and standard deviations unless stated otherwise. For nonnormally distributed data with multiple groups, we used the Kruskal–Wallis test with post hoc Dunn test with Bonferroni correction for multiple comparisons. For categorical comparison where observed frequencies were fewer than five, the Fisher exact test was used. For comparing weight changes over time, a repeated measures ANOVA was used. For investigating the role of various covariates to predict ALM (kg), we used multiple linear regression modelling using the R function ‘lm’. The model was manually fitted with clinically meaningful variables, removing variables which did not improve the model. Variables tested included sex, BMI, fat percentage, TWL, BAR vs. CON, protein intake, NAFLD score, years since surgery, diabetes, glucagon‐like peptide‐1 use, physical activity level, plasma zinc, HOMA‐IR, CRP, HbA1c and eGFR. Models were checked for normality of residuals, homoscedasticity, linearity and collinearity. Performance of the models were compared using the likelihood ratio test, and a model was retained if was *p* < 0.05 compared to the previous model. Significance was set as *p* < 0.05. All analyses were performed, and graphs were constructed using *R* Statistical Software (v4.4.1; R Core Team 2021).

## 3 Results

### 3.1 General Characteristics, Medical History and Biochemistry

**FIGURE 1:**
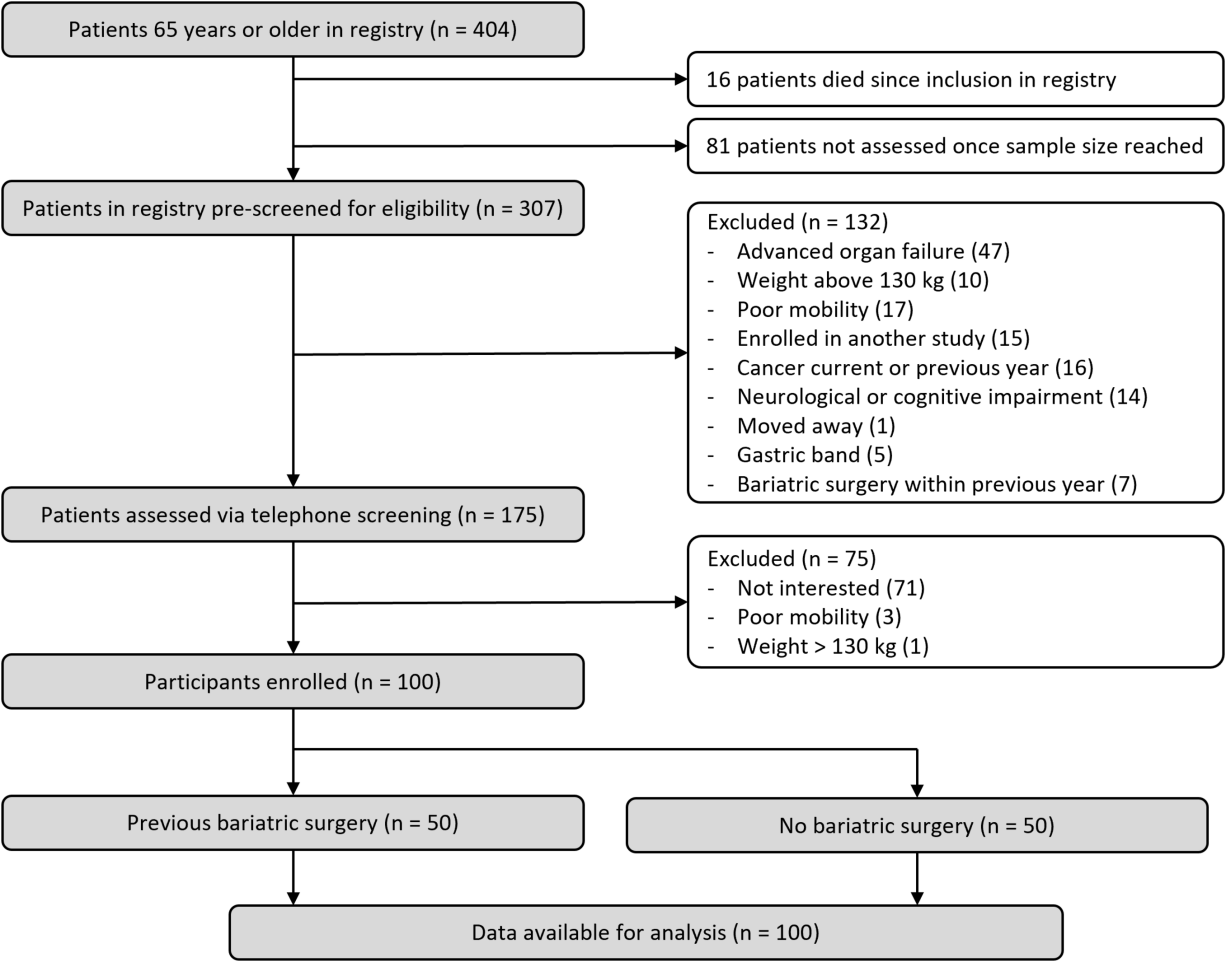
Flow diagram of study recruitment.

We screened 307 patients of which 100 patients were included, 50 for BAR and 50 for CON (Figure 1). Groups did not differ in the proportion of males included (BAR 40%, CON 35%. *p* = 0.84). BAR participants were 2.4 years younger than CON participants (68.3 ± 3.2 years vs. 70.7 ± 3.9, *p* < 0.001). BAR consisted of 25 participants after primary RYGB, 11 after secondary RYGB, 13 after SG and 1 after a vertical banded gastroplasty (Table 1). Time since surgery was 9.3 ± 5.9 years. CON had a higher prevalence of diabetes (52% vs. 28%, *p* < 0.05) and scored worse on the NAFLD‐fibrosis score than the BAR (−0.25 ± 0.97 vs. −0.74 ± 1.19, *p* < 0.05). BAR had lower levels of inflammation and insulin resistance compared to CON (CRP: 0.87 ± 1.21 mg/L vs. CON 1.86 ± 1.80, *p* < 0.01; HOMA‐IR: 2.8 ± 2.3 vs. 5.0 ± 3.9, *p* < 0.001). Both groups reported few gastrointestinal symptoms (GSRS for BAR 1.73 ± 0.77, CON 1.71 ± 0.75, *p* = 0.89).

**TABLE 1:** Patient characteristics by group.

	BAR (*n* = 50)	CON (*n* = 50)	*p*‐value
Demographic information			
Age (years)	68.3 ± 3.2	70.7 ± 3.9**	< 0.001
Sex male (%)	40%	36%	0.837
Medical history			
Number of medications	6.2 ± 3.5	6.5 ± 3.5	0.615
Diabetes prevalence (%)	28%	52%*	0.016
GLP‐1 analogue usage (%)	20%	32%	0.254
NAFLD‐fibrosis score	‐0.74 ± 1.19	−0.52 ± 0.97*	0.024
Weight loss treatment history			
Time since baseline (years)	9.2 ± 5.9	10.1 ± 15.6	0.272
Roux‐en‐Y, primary (*n*)	25		
Roux‐en‐Y, secondary (*n*)	11		
Sleeve gastrectomy (*n*)	13		
Mason gastroplasty (*n*)	1		
Biochemistry			
eGFR _Creatinine_ (mL/min/1.73m^2^)	76.4 ± 17.3	69.1 ± 15.6*	0.031
eGFR _Cystatin_ (mL/min/1.73m^2^)	94.97 ± 25.52	88.45 ± 21.40	0.170
HbA1C (mmol/mol)	40.4 ± 9.0	44.0 ± 9.0*	0.048
Vitamin D	34.5 ± 15.1	28.2 ± 11.4*	0.020
Zinc	88.9 ± 17.4	93.1 ± 12.6	0.174
CRP (mg/L)	0.87 ± 1.21	1.86 ± 1.80**	0.002
HOMA‐IR (mg/L)	2.8 ± 2.3	5.0 ± 3.9*	0.001
Sarcopenia index	81.7 ± 20.3	82.0 ± 15.3	0.939
Other			
EQ 5D5L quality of life (index score 0–1)	0.84 ± 0.14	0.75 ± 0.25*	0.022
IPAQ activity rating (MET hours/day)	5.9 ± 6.9	5.8 ± 8.6	0.950
GSRS score (1–7)	1.73 ± 0.77	1.71 ± 0.75	0.888

*Note:* Continuous data are reported as means ± standard deviations. Group comparisons with independent sample student *t*‐test.

Abbreviations: BAR, group after bariatric surgery; CON, nonsurgical group; eGFR, estimated glomerular filtration rate; EQ 5D5L, EuroQol 5‐dimensions 5‐levels questionnaire; GLP‐1, glucagon‐like peptide‐1; HbA1c, Haemoglobin A1C; IPAQ, International Physical Activity Questionnaire; METS, metabolic equivalents of task; NAFLD, nonalcoholic fatty liver disease.

* *p* < 0.05.

** *p* < 0.01.

### 3.2 Weight History

Weight, BMI, weight loss and weight regain are presented in Table 2. BAR patients had a higher BMI than CON at baseline (42.9 ± 6.2 kg/m^2^ vs. 38.2 ± 3.6, *p* < 0.001). As expected, BAR patients lost more weight after MBS than CON patients who lost weight with nonsurgical treatment (TWL 31.6 ± 9.5% vs. 12.1 ± 8.42%, *p* < 0.001). CON regained a greater proportion of lost weight compared to BAR (41.3 ± 48.7% vs. 22.4 ± 21.9, *p* < 0.05).

**TABLE 2:** Bodyweight and weight loss history by group.

	BAR(*n* = 50)	CON(*n* = 50)	*p‐*value
Height (m)	1.65 ± 0.11	1.66 ± 0.10	0.605
Weight			
Baseline (kg)	116.6 ± 19.0*	105.1 ± 12.3**	0.001
Nadir post‐treatment (kg)	79.7 ± 16.7*	92.5 ± 14.15**	< 0.001
Current (kg)	86.0 ± 19.7*	98.2 ± 13.8**	< 0.001
Body mass index			
Baseline (kg/m^2^)	42.9 ± 6.2*	38.2 ± 3.6**	0.001
Nadir post‐treatment (kg/m^2^)	29.3 ± 5.5*	33.5 ± 4.5**	< 0.001
Current (kg/m^2^)	31.5 ± 6.5*	35.6 ± 4.3**	< 0.001
Weight loss (baseline to nadir)			
Total weight loss (%)	31.6 ± 9.5	12.1 ± 8.42**	< 0.001
Excess weight loss (%)	80.4 ± 27.2	37.0 ± 27.2**	< 0.001
Weight regain			
From nadir (kg)	8.9 ± 9.6	5.8 ± 8.0	0.081
Proportion of lost weight (%)	22.4 ± 21.9	41.3 ± 48.7*	0.014

*Note:* Continuous data are reported as means ± standard deviations. Group comparisons with independent sample student *t*‐test. Groups comparisons over time points with Kruskal–Wallis's test. * *p* < 0.001 for time effect; ** *p* < 0.001 for group effect.

Abbreviations: BAR, group after bariatric surgery; CON, nonsurgical group.

### 3.3 Body Composition, Strength, Physical Performance and Physical Activity

Fat percentage was similar between CON and BAR (39.2 ± 7.8% vs. 41.6 ± 6.4, *p* = 0.09), but FFM and ALMI were lower in BAR compared to CON (FFM: 52.0 ± 12.6 kg vs. 57.5 ± 11.5, *p* < 0.05; ALMI: 7.2 ± 1.3 kg/m^2^ vs. 7.9 ± 1.4, *p* < 0.05). However, relative muscularity using %ALM/BMI was not different between the groups (BAR 64.7 ± 18.1% vs. CON 62.6 ± 15.8%, *p* = 0.53). The prevalence of low muscle mass based on the EWGSOP1 and ESPEN/EASO criteria varied between 20% and 47% based on sex and treatment group but was not statistically different for the whole group or when separated by sex (Table 3). There were no differences in SPPB score, handgrip strength, gait speed or sit‐to‐stand test between the CON and BAR. Physical activity levels were similar between groups (metabolic equivalent of task 5.9 ± 6.9 h/day vs.5.8 ± 8.6, *p* = 0.95).

**TABLE 3:** Body composition and sarcopenia assessment by group and sex.

	All participants			Males			Females		
	BAR (*n =* 50)	CON (*n =* 50)	*p*‐value	BAR (*n =* 20)	CON (*n =* 18)	*p*‐value	BAR (*n =* 30)	CON (*n =* 32)	*p*‐value
Body composition									
Fat proportion (%)	39.2 ± 7.8	41.6 ± 6.4	0.087	32.2 ± 5.5	35.0 ± 4.4	0.092	43.8 ± 5.0	45.4 ± 3.8	0.180
Obesity (%)	62%	80%	0.078	65%	89%	0.131	60%	75%	0.178
Fat free mass (kg)	52.0 ± 12.6	57.5 ± 11.5	0.024*	63.4 ± 9.5	69.2 ± 8.4	0.056	44.4 ± 7.7	51.0 ± 6.7	< 0.001**
ALM (kg)	20.0 ± 5.6	22.1 ± 5.4	0.064	25.1 ± 4.4	27.4 ± 4.3	0.106	16.7 ± 3.4	19.1 ± 3.2	0.005*
ALMI (kg/m^2^)	7.2 ± 1.3	7.9 ± 1.4	0.012*	8.1 ± 1.2	8.9 ± 1.1	0.040	6.7 ± 1.2	7.4 ± 1.2	0.018*
Skeletal muscle mass (kg)	21.8 ± 6.3	24.1 ± 6.0	0.064*	27.4 ± 4.9	30.1 ± 4.8	0.106	18.1 ± 3.8	20.8 ± 3.5	0.005*
%ALM/BMI	64.7 ± 18.7	62.6 ± 15.8	0.530	82.8 ± 12.7	79.3 ± 11.9	0.382	52.6 ± 8.3	53.2 ± 7.9	0.793
Low muscle mass									
EWGSOP1 (%)	42%	34%	0.537	35%	33%	1.000	47%	34%	0.467
ESPEN/EASO (%)	26%	34%	0.512	35%	56%	0.344	20%	22%	1.000
Strength									
Handgrip (kg)	33.9 ± 10.5	34.6 ± 10.8	0.757	42.9 ± 9.5	44.7 ± 9.6	0.573	27.9 ± 5.7	28.9 ± 6.4	0.533
Poor sit‐to‐stand time (%)	16%	20%	0.795	10%	17%	0.900	20%	22%	1.000
Physical performance									
SPPB score (0–12)	10.4 ± 2.0	10.3 ± 2.0	0.800	10.7 ± 1.8	11.1 ± 1.3	0.376	10.2 ± 2.1	9.8 ± 2.1	0.472
Gait speed (m/s)	0.95 ± 0.22	0.97 ± 0.23	0.532	1.01 ± 0.21	1.06 ± 0.18	0.475	0.90 ± 0.23	0.93 ± 0.25	0.670
Sarcopenia									
Sarcopenia (%)	2%	4%	1.000	5%	0%	1.000	0%	6%	0.492
Sarcopenic obesity (%)	4%	14%	0.160	5%	17%	0.328	3%	13%	0.356

*Note:* Continuous data are reported as means ±SD. Group comparisons with independent sample student *t*‐test for parametric variable and Mann–Whitney *U*‐test for nonparametric variables. Categorical group comparisons with Chi‐square test or with Fisher's exact test when expected frequency < 5.

Abbreviations: ALM, appendicular lean mass; ALMI, appendicular lean mass index; BAR, group after bariatric surgery; BMI, body mass index; CON, nonsurgical group; SPPB, short physical performance battery.

* *p* < 0.05.

** *p* < 0.001.

### 3.4 Sarcopenia and Sarcopenic Obesity Prevalence

The EWGSOP1 criteria for sarcopenia was only met in 3% of the total study population, and the ESPEN/EASO sarcopenic obesity prevalence was 9%. Although sarcopenic obesity prevalence was 15% in CON and 4% in BAR, this was not significant (*p* = 0.159). Sarcopenia index was not different between the groups (81.7 ± 20.3 vs. 82.0 ± 15.3, *p* = 0.939).

### 3.5 Dietary Intake With Emphasis on Protein

Energy, fat and carbohydrate intakes were similar between the BAR and CON (Table 4). Dietary intake for males was 1875 ± 480 kcal/day and for females was 1420 ± 321 kcal/day. For comparison, REE was 1761 ± 198 kcal/day for males and 1357 ± 189 kcal/day for females. Protein intake per kilogramme actual bodyweight was higher for BAR than for CON (0.83 ± 0.25 g/kg/day vs. 0.73 ± 18, *p* < 0.05). This difference remained as a tendency when expressed relative to FFM (BAR 1.36 ± 0.36 g/kg/day FFM vs. CON 1.25 ± 0.27, *p* = 0.09).

**TABLE 4:** Dietary intake by group.

	BAR (*n* = 50)	CON (*n* = 50)	*p*‐value
Energy intake			
Total daily intake (kcal)	1524.0 ± 453.3	1662.3 ± 432.9	0.122
Energy/IBW (kcal/kg)	19.8 ± 6.4	19.5 ± 6.3	0.807
Fat intake			
Total daily intake (g)	61.3 ± 24.2	70.6 ± 24.6	0.059
Fat as % energy	35.6 ± 7.1	37.8 ± 8.1	0.164
Carbohydrate intake			
Total daily intake (g)	154.8 ± 40.9	166.6 ± 54.1	0.221
Carbohydrate as % energy	41.3 ± 6.3	40.3 ± 7.9	0.490
Total sugar (g)	65.0 ± 26.2	65.1 ± 35.3	0.995
Fibre intake (g)	15.1 ± 5.0	16.4 ± 6.1	0.230
Protein intake			
Total daily intake (g)	69.8 ± 21.6	71.5 ± 19.4	0.672
Protein as % energy	18.6 ± 4.1	17.5 ± 3.3	0.138
Protein/BW (g/kg)	0.83 ± 0.25	0.73 ± 18*	0.029
Protein/IBW (g//kg)	0.90 ± 0.29	0.84 ± 0.29	0.313
Protein/FFM (g/kg)	1.36 ± 0.36	1.25 ± 0.27	0.088

*Note:* Continuous data are reported as means ± standard deviations. Group comparisons with independent sample student *t*‐test.

Abbreviations: BAR, group after bariatric surgery; BW, body weight; CON, nonsurgical group; FFM, fat free mass; IBW, ideal body weight.

* *p* < 0.05.

**FIGURE 2:**
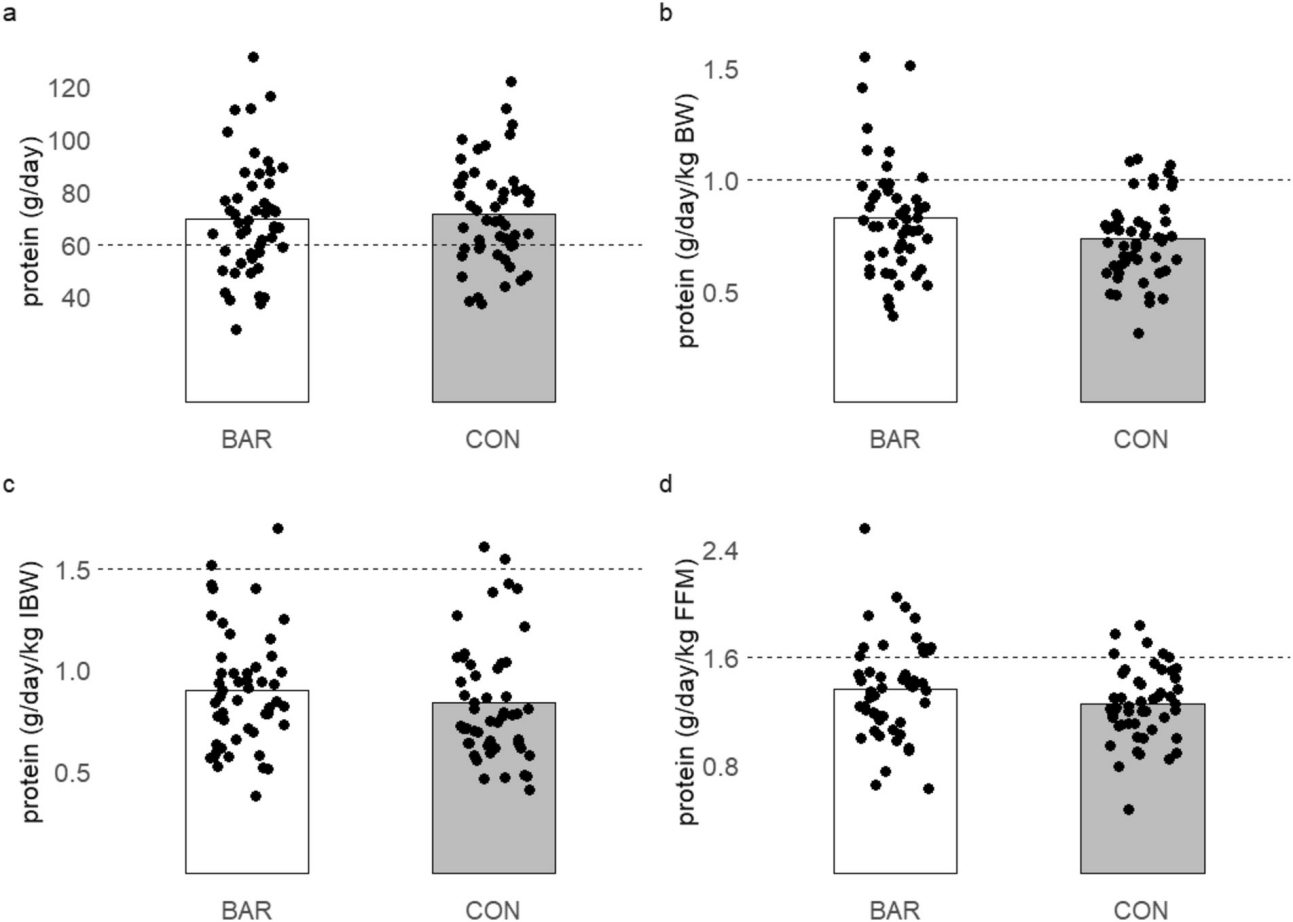
Protein intake by group compared to protein recommendations. All bars are mean for the groups. (a) Total dietary intake compared to minimum recommendations after MBS of 60 g/day. (b) Intake/actual bodyweight (ABW) compared to recommended intake for older adults of > 1 g/kg/day. (c) Intake/ideal bodyweight (IBW) compared to recommended intakes of 1.5 g/kg/IBW recommendation after MBS. (d) Intake/fat free mass compared to EAR of 1.6 g/kg FFM for older adults determined by the indicator amino acid oxidation (IAAO) method.

For BAR, 32% of participants did not meet the minimum intake level of 60 g of protein/day and only 4% met the 1.5 g/kg IBW recommended after MBS (Figure 2) [28]. When comparing to recommendations of 1 g/kg/BW for older adults, this was achieved by 13% of the total population based on actual body weight [29]. Similarly, only 19% of all patients achieved 1.6 g/kg FFM recommended for older adults [27].

### 3.6 Quality of Life

HRQoL index score was higher for BAR than for CON (0.84 ± 0.14 vs. 0.75 ± 0.25, *p* < 0.05). Self‐rated health (BAR 75.62 ± 16.71 vs. CON 75.84 ± 12.94, *p* = 0.941) and the percentage of participants with optimal health (BAR 23% vs. CON 20%, *p* = 0.635) were not different between groups.

### 3.7 Factors Predicting ALM

**FIGURE 3:**
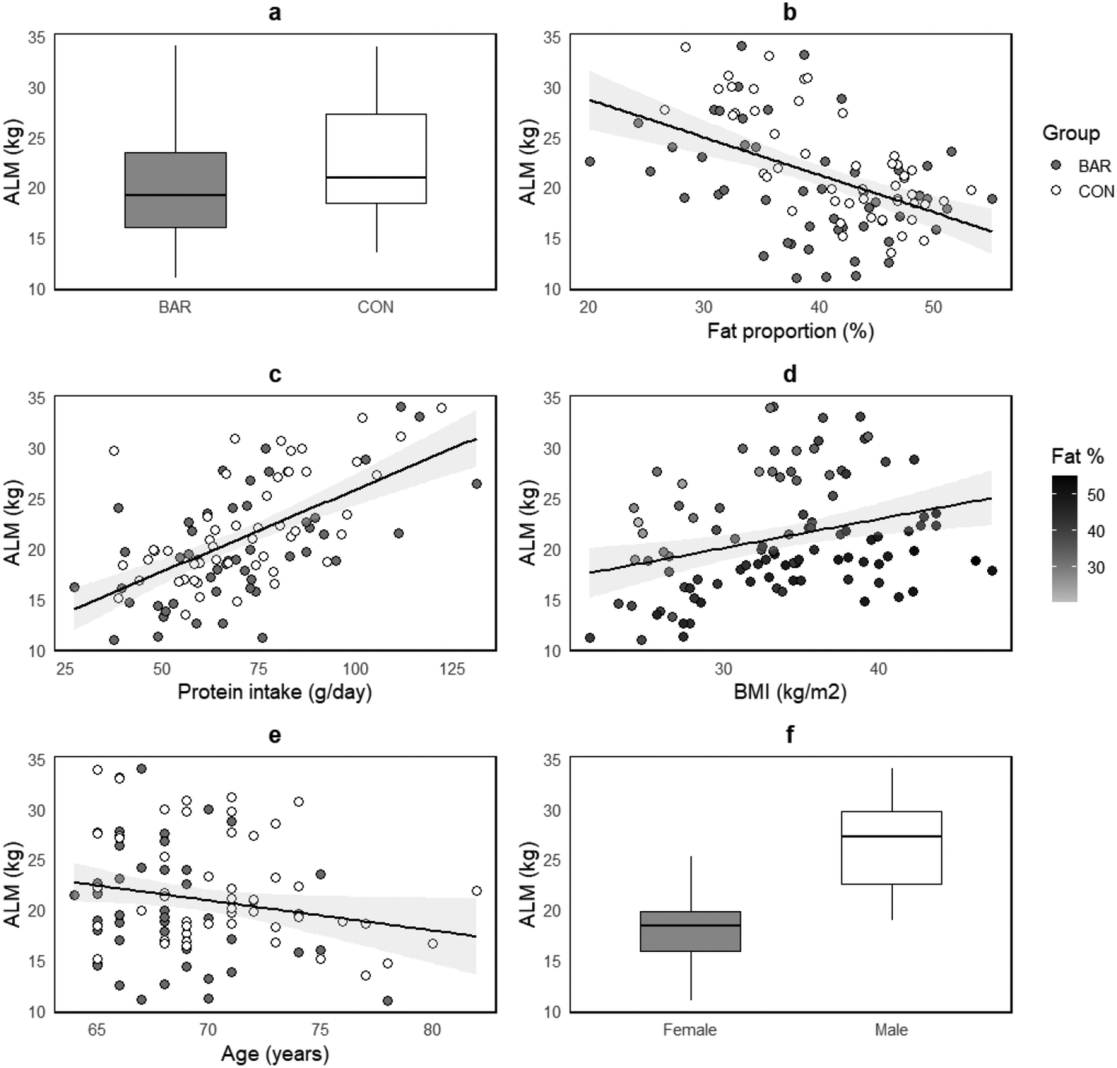
Linear model effects on appendicular lean mass (ALM) sex. BAR vs. CON did not improve model fit but is included visually for comparison purposes. Shaded grey areas are 95% confidence intervals. Plot d depicts the effect of BMI on the *x*‐axis and the interaction effect between BMI and fat proportion through shading of data points.

In the linear regression model, male sex was associated with a 4.39 kg higher ALM compared to females (*p* < 0.001). Each unit increase in BMI corresponded to a 1.48 kg increase in ALM (*p* < 0.001), while each additional year of age was linked to a 0.19 kg decline (*p* = 0.007). Fat percentage showed a positive but nonsignificant association with ALM (0.37 kg, *p* = 0.061), but it significantly interacted with BMI, indicating that as BMI increases, the positive effect of fat percentage on ALM diminishes (−0.02 kg, *p* < 0.001). Each additional gramme of dietary protein per day predicted a 0.04 kg increase in ALM (*p* = 0.008), while BAR compared to CON did not improve prediction. The model explained a substantial proportion of the variance in ALM (Adjusted *R* ^2^ = 0.8035, *p* < 0.001; Table 5 and Figure 3).

**TABLE 5:** Multiple linear regression models fitted for appendicular lean mass (kg).

	Estimate (95% CI)	*p*‐value
Intercept	−5.045 (8.886, −0.568)	
Sex (male)	4.387 (0.898, 4.884)	< 0.001
BMI (kg/m^2^)	1.478 (0.252, 5.866)	< 0.001
Fat percentage (%)	−0.021 (0.006, −3.761)	0.061
BMI*fat percentage	−0.021 (0.006, −3.761)	< 0.001
Age (years)	−0.193 (0.070, −2.751)	0.007
Protein intake (g/day)	0.040 (0.015, 2.709)	0.008

*Note:* Adjusted *R*‐squared = 0.8035, *p* < 0.001.

Abbreviations: %ALM/BMI, %appendicular lean mass/body mass index; CI, confidence interval; CON, nonsurgical group; SD, standard deviation.

## 4 Discussion

Our study aimed to investigate sarcopenia and muscle mass in older adults after MBS by comparing them to a nonsurgical group. The prevalence of sarcopenia was low across groups at 3% overall. Given this low prevalence, the comparison of sarcopenia between groups was not meaningful. We used EWSGOP1 criteria to define sarcopenia as the updated EWSGOP2 criteria do not make provision for obesity within its appendicular mass cut‐offs. Indeed, several studies have critiqued the EWSGPO2 guidelines as less sensitive, including for MBS or obesity [7, 30, 31, 32]. A recent systematic review suggested that EWSGOP1 maybe be better at predicting poor health outcomes than EWSGOP2 [33]. For people with obesity, it is important to use muscle mass and strength indices which consider the extra muscle mass accrued with obesity. Only the EWSGOP1 guidelines provide handgrip cut‐off points per BMI category. However, even using these cut‐offs, only 5% of our sample population had low handgrip. Ruthes et al. recently argued that low muscle mass is a more important indicator of sarcopenia after MBS rather than muscle strength, which contrasts with updated sarcopenia guidelines [31]. Most sarcopenia research after MBS has been conducted in adults under 65 years, and sarcopenia guidelines were not developed for this purpose. Therefore, while we agree that the emphasis on muscle quantity is more important in nonolder adults, this should be termed *presarcopenia* if muscle mass is the defining criterion. Our study is the first report of muscle mass and sarcopenia exclusively in older adults after MBS. Still, 90% of participants were young‐old (65 to 74 years), and none were oldest‐old (>85 yeas). This may have contributed to the low prevalence of sarcopenia, as prevalence increases with age. As it remains important to identify presarcopenia after MBS, universal cut‐offs, adjusted for adiposity, are needed.

The ESPEN/EASO sarcopenic obesity prevalence was 9% even though prevalence of obesity was high (>60% by group and sex); therefore, the low prevalence of sarcopenic obesity is mainly due to the sarcopenia criterium not being met. A recent study by Vieira et al. investigated sarcopenic obesity in 186 participants at least 2 years after MBS [7]. They reported 8% ESPEN/EASO sarcopenic obesity when using bioimpedance analysis (BIA) and 23% using DXA. When using EWSGOP2 to define the sarcopenia component, this was only 1% for DXA. The participants in this study were much younger (mean 44 years) compared to ours, and only 10% were male. Another recent study reported 13%–23% ESPEN/EASO sarcopenic obesity in 124 candidates before bariatric surgery, all adults younger than 60 years [8]. A third recent study reported ESPEN/EASO sarcopenic obesity prevalence of 89% in nonolder women presurgery, dramatically decreasing to 3% at 12 months after surgery [9]. They did not include strength or physical performance tests, which means sarcopenic obesity in their results are in fact presarcopenia. The dramatic drop in low muscle mass illustrates the importance of selecting appropriate cut‐offs and interpreting them correctly. This study used ALM/weight which improved with dramatic weight loss. However, this could provide a skewed picture as absolute ALM decreased by approximately 4 kg. A recent critical review stresses the importance of selecting sarcopenia criteria which are suitable to the study objective [34].

When comparing our findings with those of older adults without MBS, the prevalence of sarcopenic obesity appears modest. A recent study of 90 patients with at least class II obesity and older than 60 years reported an ESPEN/EASO sarcopenic obesity prevalence of 23%–40%, dependant on diagnostic cut‐offs used [35]. In a larger study with 1559 participants who were older (mean 75 years) and did not all have obesity, ESPEN/EASO sarcopenic obesity was 30%. In a third study, in patients with obesity and above 70 years, prevalence was 27%, based on BIA assessments [36]. As our study investigated the role of MBS in sarcopenia, we excluded other potential causes of sarcopenia, and this could explain some of these differences. Unlike the Global Leadership in Malnutrition (GLIM) criteria for malnutrition, sarcopenia criteria do not include an aetiology component, and it is applied to ageing populations and populations with a specific disease, irrespective of age [37]. Separating the role of age and disease in sarcopenia will be complex, but aetiology should be considered, and cut‐offs should be age‐dependant.

With several definitions of both sarcopenia and obesity and several cut‐off points even within definitions, it will remain difficult to compare studies until more standardized methodologies are agreed upon. In the interim, our findings on muscle mass rather than sarcopenia are more clinically relevant. When considering the higher BMI of the CON group, there was no difference in muscle mass between the groups. Similarly, there were no differences in any measure of strength or physical performance between the two groups. Despite recruiting participants from the same clinic, the groups had important differences. For example, the BAR group had a higher baseline BMI, more weight loss, lower diabetes prevalence, were 2 years younger, had less systemic inflammation and fewer had MAFLD. In fact, several of these factors may explain why BAR participants were not more prone to low muscle mass than CON patients. The lack of difference in muscle mass between the two groups was also confirmed by the similarity in the sarcopenic index biomarker between the groups.

A recent meta‐analysis demonstrated that absolute FFM and muscle mass decrease when comparing patients prior to and after bariatric surgery [5]. However, whether the amount of muscle mass loss is excessive when taking the change in total body weight into account is more clinically relevant. Our study shows that muscle mass relative to BMI is not different for BAR compared to CON. The regression model we fitted confirmed this across the whole study population, indicating that sex, BMI, fat percentage and age were important predictors of muscle mass. The impact of these factors is expected but nonetheless important to illustrate that these factors predict muscle while MBS does not. Overall, the absence of lower muscle mass in the BAR group is reassuring, indicating that older adults with previous MBS can have equal if not better musculoskeletal function than nonoperated individuals.

In a study with a similar design to ours, Santini et al. matched 41 postmenopausal women who previously underwent RYGB surgery (mean 7.5 years prior) with 41 women with obesity. ALM adjusted for BMI, and age was not different between the groups, and TWL was associated with change in lean mass in multiple linear regression analysis [38]. This contrasts with the study from Buzza et al., who compared postmenopausal women at least 2 years after RYGB with an age‐matched control group and reported lower ALMI in the RYGB group. However, in the latter study, the RYGB group had a lower BMI, yet ALMI was not adjusted for BMI or weight [6]. Still, neither of these studies includes adults older than 65 years.

A secondary aim of our study was to investigate the role of protein intake in maintaining muscle mass after MBS. Clinical guidelines recommend 60 g protein per day, but this singular value does not account for differences in body size between individuals. Guidelines mention a secondary protein target of up to 1.5 g/day/kg IBW [28]. While 68% of the BAR group achieved 60 g/day, only 4% met the second target. Inadequate protein intake after MBS is well‐documented; however, our results pertain to older adults, who are more vulnerable to sarcopenia [10]. Though many patients did not meet recommendations, it is encouraging that protein intake was not lower for BAR than for CON. We also compared protein intake against recommended intake for older adults of at least 1.0 g/kg, but these recommendations do not make provision for obesity and therefore may underestimate recommended intake [26]. Therefore, we compared the protein intake to protein requirements of 1.6 g/day/kg FFM which were determined for older adults using the Indicator Amino Acid Oxidation method (IAAO) method [27]. Only 19% met this target, with no group differences. In our regression model, protein intake was an independent predictor of muscle mass, although the effect size was small. Still, this suggests that low protein intake could contribute to presarcopenia, predisposing individuals to sarcopenia as they age. This finding underscores the need for continued screening for presarcopenia, including assessment for and dietary modification of low protein intake. Our findings also highlight the need for accurate protein recommendations after MBS.

### 4.1 Limitations

We powered our study to measure differences in muscle mass between two groups, and therefore, we are confident that the lack of difference between groups, confirmed by our regression model, shows that individuals with previous MBS are not at greater risk of low muscle mass compared to patients with obesity treated nonsurgically. However, the sample size remains small, and subanalysis by sex, surgery type or health state is underpowered and should be considered exploratory in nature. The sample size of 100 further restricts the number of variables that can be included in the multiple linear regression analysis, potentially limiting the complexity of the model. Equally, the prevalence of sarcopenia was very low, making comparison between groups impossible. Sarcopenic obesity was numerically higher in CON, and it is possible that the difference could grow towards significance in a larger sample.

We recruited 100 participants after excluding 132 patients during prescreening based on their poor health and removing 16 patients who had died. If proportionately more patients with or without MBS were excluded, the remaining sample could be biassed. Such bias could be bidirectional as BAR participants have more severe obesity at baseline, yet they lose more weight and may thus have greater improvements in metabolic health thereafter. However, exclusion based on factors such as advanced organ failure, poor mobility or cancer was deemed important to exclude other causes of sarcopenia. Additionally, as all participants were from one region in Belgium, the generalizability of the findings to other populations could be questioned. Still, our study is the first to study muscle mass specifically in older adults after MBS and therefore remains valuable.

The cross‐sectional design restricts inferences to causality. To address this to some extent, we included retrospective weight histories for both groups and investigated these in regression modelling. Yet, future prospective studies are warranted to confirm our findings.

A final limitation of our study is the use of self‐reported dietary intake methodology, namely, 24‐h recalls. Comparison of energy intake and estimated REE suggests that dietary energy intake is an underestimation, although the older age, low activity levels and high adiposity of our sample will contribute to lower total energy expenditure (TEE). A substantial body of literature shows that self‐reported energy intake is underreported in people with obesity, irrespective of measurement tool used [39]. Such bias is difficult to avoid in nutritional studies, yet comparison with biomarkers previously showed that underreporting for protein is typically less than for energy. The use of two versus one 24‐h recalls further improves the prediction of true protein intake [40]. Nitrogen excretion can be used to estimate protein intake, although faecal nitrogen excretion changes after MBS, potentially limiting the applicability of this method [41].

## 5 Conclusion

In summary, our study suggests that older adults with previous MBS are not more likely to develop sarcopenia than older people for whom obesity was nonsurgically treated. Rather, older age, higher adiposity and lower protein intake can lead to lowered muscle mass and thereby predispose patients to sarcopenia. Standardized methodologies to assess sarcopenic obesity which also takes aetiology and age into account will enable the comparison of studies and aid in evaluating interventions to treat sarcopenia.

## Author Contributions

Gabriël Eksteen, Bart Van der Schueren, Tim Vanuytsel and Christophe Matthys conceptualized the study. Gabriël Eksteen conducted study assessments and wrote the initial manuscript draft. All authors critically reviewed and edited the manuscript. All authors approved the final version of the manuscript.

## Ethics Statement

The authors certify that the manuscript complies with the ethical guidelines for authorship and publishing in the Journal of Cachexia, Sarcopenia and Muscle. The study was reviewed and approved by the research ethical committee of UZ Leuven hospital and KU Leuven University (s66815). The study was conducted in compliance with the principles of the Declaration of Helsinki, the principles of Good Clinical Practice (GCP) and fulfilled all the applicable regulatory requirements. The study was registered with clinicaltrials.gov (NCT05582668). All patients signed informed consent to participate in the study.

## Conflicts of Interest

The authors declare no conflicts of interest.

## Data Availability

The raw data supporting the conclusions of this article are available in the KU Leuven repository RDR (https://doi.org/10.48804/YHM3J7). The data are not openly available for ethical reasons, but access can be granted on request by the authors.

## References

[1] WHO European Regional Obesity Report 2022. Copenhagen: WHO Regional Office for Europe, (2022).

[2] L. M. Donini , L. Busetto , S. C. Bischoff , et al., “Definition and Diagnostic Criteria for Sarcopenic Obesity: ESPEN and EASO Consensus Statement,” Obesity Facts 15, no. 3 (2022): 321–335. 35196654 10.1159/000521241 PMC9210010

[3] A. Kalinkovich and G. Livshits , “Sarcopenic Obesity or Obese Sarcopenia: A Cross Talk Between age‐Associated Adipose Tissue and Skeletal Muscle Inflammation as a Main Mechanism of the Pathogenesis,” Ageing Research Reviews 35 (2017): 200–221. 27702700 10.1016/j.arr.2016.09.008

[4] J. Liao , Y. Yin , J. Zhong , et al., “Bariatric Surgery and Health Outcomes: An Umbrella Analysis,” Frontiers in Endocrinology 13 (2022): 1016613. 36387921 10.3389/fendo.2022.1016613 PMC9650489

[5] M. A. H. Nuijten , T. M. H. Eijsvogels , B. Sanders , et al., “Changes in Fat‐Free Mass, Protein Intake and Habitual Physical Activity Following Roux‐En‐Y Gastric Bypass Surgery: A Prospective Study,” Obesity Surgery 33, no. 7 (2023): 2148–2157. 37249699 10.1007/s11695-023-06650-y PMC10228447

[6] A. F. B. Buzza , C. A. Machado , F. Pontes , et al., “Prevalence of Sarcopenia in Women at Stable Weight Phase After Roux‐En‐Y Gastric Bypass,” Archives of Endocrinology and Metabolism 66 no. 3 (2022): 362–371. 35657128 10.20945/2359-3997000000494 PMC9832848

[7] F. T. Vieira , K. Godziuk , F. Lamarca , et al., “Sarcopenic Obesity Diagnosis by Different Criteria Mid‐to Long‐Term Post‐Bariatric Surgery,” Clinical Nutrition 41, no. 9 (2022): 1932–1941. 35947895 10.1016/j.clnu.2022.07.006

[8] E. González Arnáiz , D. Ariadel Cobo , B. Estébanez , et al., “Prevalence of Sarcopenic Obesity According to Different Diagnostic Methods and Cut‐Off Points in Candidates for Bariatric Surgery,” Clinical Nutrition 43, no. 5 (2024): 1087–1093. 10.1016/j.clnu.2024.03.015 38579371

[9] P. S. Rodrigues , F. M. Mendonça , J. S. Neves , et al., “Effects of Bariatric Surgery on Sarcopenic Obesity Outcomes: A One‐Year Prospective Study in Middle‐Aged Women,” Obesity Surgery 34, no. 5 (2024): 1674–1683. 38523172 10.1007/s11695-024-07164-x

[10] N. Steenackers , I. Gesquiere , and C. Matthys , “The Relevance of Dietary Protein After Bariatric Surgery: What Do We Know?,” Current Opinion in Clinical Nutrition and Metabolic Care 21, no. 1 (2018): 58–63. 29035973 10.1097/MCO.0000000000000437

[11] J. R. Villarreal‐Calderon , R. Cuellar‐Tamez , E. C. Castillo , E. Luna‐Ceron , G. García‐Rivas , and L. Elizondo‐Montemayor , “Metabolic Shift Precedes the Resolution of Inflammation in a Cohort of Patients Undergoing Bariatric and Metabolic Surgery,” Scientific Reports 11, no. 1 (2021): 12127. 34108550 10.1038/s41598-021-91393-y PMC8190106

[12] E. Von Elm , D. G. Altman , M. Egger , S. J. Pocock , P. C. Gøtzsche , and J. P. Vandenbroucke , “The Strengthening the Reporting of Observational Studies in Epidemiology (STROBE) Statement: Guidelines for Reporting Observational Studies,” Lancet 370, no. 9596 (2007): 1453–1457. 18064739 10.1016/S0140-6736(07)61602-X

[13] S. S. Park , S. Lim , H. Kim , and K. M. Kim , “Comparison of two DXA Systems, Hologic Horizon W and GE Lunar Prodigy, for Assessing Body Composition in Healthy Korean Adults,” Endocrinology and Metabolism 36, no. 6 (2021): 1219–1231. 34911173 10.3803/EnM.2021.1274 PMC8743584

[14] C. McCarthy , G. M. Tinsley , A. Bosy‐Westphal , et al., “Total and Regional Appendicular Skeletal Muscle Mass Prediction From Dual‐Energy X‐Ray Absorptiometry Body Composition Models,” Scientific Reports 13, no. 1 (2023): 2590. 36788294 10.1038/s41598-023-29827-y PMC9929067

[15] P. M. Cawthon , K. W. Peters , M. D. Shardell , et al., “Cutpoints for low Appendicular Lean Mass That Identify Older Adults With Clinically Significant Weakness,” Journals of Gerontology Series A: Biological Sciences and Medical Sciences 69, no. 5 (2014): 567–575. 24737559 10.1093/gerona/glu023 PMC3991141

[16] M. L. Macena , A. E. Silva, Jr. , J. M. Melo , D. T. Paula , D. R. S. Praxedes , and N. B. Bueno , “Estimates of Resting Energy Expenditure and Total Energy Expenditure Using Predictive Equations for Individuals After Bariatric Surgery: A Systematic Review With Meta‐Analysis,” Obesity Surgery 33 no. 12 (2023): 3999–4006. 37889369 10.1007/s11695-023-06908-5

[17] A. Drolz , S. Wolter , M. H. Wehmeyer , et al., “Performance of Non‐Invasive Fibrosis Scores in Non‐Alcoholic Fatty Liver Disease With and Without Morbid Obesity,” International Journal of Obesity 45, no. 10 (2021): 2197–2204. 34168277 10.1038/s41366-021-00881-8 PMC8455320

[18] T. J. Wilkinson , L. A. Baker , E. L. Watson , A. C. Smith , and T. Yates , “Diagnostic Accuracy of a ‘Sarcopenia Index’ Based on Serum Biomarkers Creatinine and Cystatin C in 458,702 UK Biobank Participants,” Clinical Nutrition ESPEN 63 (2024): 207–213. 38968079 10.1016/j.clnesp.2024.06.041

[19] C. L. Craig , A. L. Marshall , M. Sjöström , et al., “International Physical Activity Questionnaire: 12‐Country Reliability and Validity,” Medicine & Science in Sports & Exercise 35, no. 8 (2003): 1381–1395. 12900694 10.1249/01.MSS.0000078924.61453.FB

[20] J. Svedlund , I. SjöDin , and G. Dotevall , “GSRS?A Clinical Rating Scale for Gastrointestinal Symptoms in Patients With Irritable Bowel Syndrome and Peptic Ulcer Disease,” Digestive Diseases and Sciences 33, no. 2 (1988): 129–134. 3123181 10.1007/BF01535722

[21] N. Bouckaert , I. Cleemput , S. Devriese , and S. Gerkens , “An EQ‐5D‐5L Value Set for Belgium,” PharmacoEconomics ‐ Open 6, no. 6 (2022): 823–836. 35927410 10.1007/s41669-022-00353-3 PMC9362639

[22] J. M. Guralnik , E. M. Simonsick , L. Ferrucci , et al., “A Short Physical Performance Battery Assessing Lower Extremity Function: Association With Self‐Reported Disability and Prediction of Mortality and Nursing Home Admission,” Journal of Gerontology 49, no. 2 (1994): M85–M94. 8126356 10.1093/geronj/49.2.m85

[23] H. C. Roberts , H. J. Denison , H. J. Martin , et al., “A Review of the Measurement of Grip Strength in Clinical and Epidemiological Studies: Towards a Standardised Approach,” Age and Ageing 40, no. 4 (2011): 423–429. 21624928 10.1093/ageing/afr051

[24] A. J. Cruz‐Jentoft , J. P. Baeyens , J. M. Bauer , et al., “Sarcopenia: European Consensus on Definition and Diagnosis,” Age and Ageing 39, no. 4 (2010): 412–423. 20392703 10.1093/ageing/afq034 PMC2886201

[25] A. J. Moshfegh , D. G. Rhodes , D. J. Baer , et al., “The US Department of Agriculture Automated Multiple‐Pass Method Reduces Bias in the Collection of Energy Intakes,” American Journal of Clinical Nutrition 88, no. 2 (2008): 324–332. 18689367 10.1093/ajcn/88.2.324

[26] J. Bauer , G. Biolo , T. Cederholm , et al., “Evidence‐Based Recommendations for Optimal Dietary Protein Intake in Older People: A Position Paper From the PROT‐AGE Study Group,” Journal of the American Medical Directors Association 14, no. 8 (2013): 542–559. 23867520 10.1016/j.jamda.2013.05.021

[27] M. Rafii , K. Chapman , R. Elango , et al., “Dietary Protein Requirement of Men >65 Years Old Determined by the Indicator Amino Acid Oxidation Technique Is Higher than the Current Estimated Average Requirement,” Journal of Nutrition 146, no. 4 (2016): 681–687. 10.3945/jn.115.225631 26962173

[28] J. I. Mechanick , C. Apovian , S. Brethauer , et al., “Clinical Practice Guidelines for the Perioperative Nutrition, Metabolic, and Nonsurgical Support of Patients Undergoing Bariatric Procedures – 2019 Update: Cosponsored by American Association of Clinical Endocrinologists/American College of Endocrinology,” Obesity 28, no. 4 (2020): O1–O58. 32202076 10.1002/oby.22719

[29] Y. Nishimura , G. Højfeldt , L. Breen , I. Tetens , and L. Holm , “Dietary Protein Requirements and Recommendations for Healthy Older Adults: A Critical Narrative Review of the Scientific Evidence,” Nutrition Research Reviews 36, no. 1 (2023): 69–85. 34666855 10.1017/S0954422421000329

[30] D. Scott , F. Blyth , V. Naganathan , et al., “Sarcopenia Prevalence and Functional Outcomes in Older men With Obesity: Comparing the use of the EWGSOP2 Sarcopenia Versus ESPEN‐EASO Sarcopenic Obesity Consensus Definitions,” Clinical Nutrition 42, no. 9 (2023): 1610–1618. 37481869 10.1016/j.clnu.2023.07.014

[31] E. M. Ruthes , B. C. Lenardt , A. D. Lass , et al., “Lean Mass and Strength Profile of Women Submitted to Bariatric Surgery: Comparison of the EWGSOP2 and FNIH Classification for Sarcopenia–ASBS Program Phase II,” Gynecological Endocrinology 38, no. 10 (2022): 868–873. 36067795 10.1080/09513590.2022.2119956

[32] E. Ramirez , R. Salas , C. Bouzas , R. Pastor , and J. A. Tur , “Comparison Between Original and Reviewed Consensus of European Working Group on Sarcopenia in Older People: A Probabilistic Cross‐Sectional Survey Among Community‐Dwelling Older People,” Gerontology 68, no. 8 (2022): 869–876. 34592734 10.1159/000519304

[33] L. V. Fernandes , A. E. G. Paiva , A. C. B. Silva , et al., “Prevalence of Sarcopenia According to EWGSOP1 and EWGSOP2 in Older Adults and Their Associations With Unfavorable Health Outcomes: A Systematic Review,” Aging Clinical and Experimental Research 34, no. 3 (2022): 505–514. 34398438 10.1007/s40520-021-01951-7

[34] G. Voulgaridou , S. Tyrovolas , P. Detopoulou , et al., “Diagnostic Criteria and Measurement Techniques of Sarcopenia: A Critical Evaluation of the Up‐to‐Date Evidence,” Nutrients 16, no. 3 (2024): 436. 38337720 10.3390/nu16030436 PMC10856900

[35] A. L. Danielewicz , A. Marra , G. Tringali , et al., “Analysis of Sarcopenic Obesity Prevalence and Diagnostic Agreement According to the 2022 ESPEN and EASO Consensus in Hospitalized Older Adults With Severe Obesity,” Frontiers in Endocrinology 15 (2024): 1366229. 38966224 10.3389/fendo.2024.1366229 PMC11222587

[36] R. Cancello , E. Brenna , D. Soranna , et al., “Sarcopenia Prevalence Among Hospitalized Patients With Severe Obesity: An Observational Study,” Journal of Clinical Medicine 13, no. 10 (2024): 2880. 38792422 10.3390/jcm13102880 PMC11122386

[37] T. Cederholm , G. Jensen , M. Correia , et al., “GLIM Criteria for the Diagnosis of Malnutrition—A Consensus Report From the Global Clinical Nutrition Community,” Journal of Cachexia, Sarcopenia and Muscle 10, no. 1 (2019): 207–217. 30920778 10.1002/jcsm.12383 PMC6438340

[38] S. Santini , N. Vionnet , J. Pasquier , et al., “Long‐Term Body Composition Improvement in Post‐Menopausal Women Following Bariatric Surgery: A Cross‐Sectional and Case–Control Study,” European Journal of Endocrinology 186, no. 2 (2022): 255–263. 34879003 10.1530/EJE-21-0895 PMC8789027

[39] H. Wehling and J. Lusher , “People With a Body Mass Index ⩾ 30 Under‐Report Their Dietary Intake: A Systematic Review,” Journal of Health Psychology 24, no. 14 (2019): 2042–2059. 28810493 10.1177/1359105317714318

[40] L. S. Freedman , J. M. Commins , J. E. Moler , et al., “Pooled Results From 5 Validation Studies of Dietary Self‐Report Instruments Using Recovery Biomarkers for Energy and Protein Intake,” American Journal of Epidemiology 180, no. 2 (2014): 172–188. 24918187 10.1093/aje/kwu116 PMC4082341

[41] C. Evenepoel , G. Vandermeulen , A. Luypaerts , et al., “The Impact of Bariatric Surgery on Macronutrient Malabsorption Depends on the Type of Procedure,” Frontiers in Nutrition 9 (2023): 1028881. 36712518 10.3389/fnut.2022.1028881 PMC9877414

